# Therapeutic Implications of Phenolic Acids for Ameliorating Inflammatory Bowel Disease

**DOI:** 10.3390/nu16091347

**Published:** 2024-04-29

**Authors:** Yanan Lu, Xue Han

**Affiliations:** 1 School of Biomedicine, Beijing City University, Huanghoudian Village, Yongfeng Town, Haidian District, Beijing 100094, China; nan0914@126.com; 2 Department of Physiology and Pathophysiology, School of Basic Medical Sciences, State Key Laboratory of Vascular Homeostasis and Remodeling, Peking University, Beijing 100191, China

**Keywords:** phenolic acids, inflammatory bowel disease, oxidative stress, immune, gut microbes

## Abstract

Inflammatory bowel disease (IBD) is a chronic inflammatory intestinal disorder, and its complex etiology makes prevention and treatment challenging. Research on new drugs and treatment strategies is currently a focal point. Phenolic acids are widely present in plant-based diets and have demonstrated the potential to alleviate colitis due to their powerful antioxidant and anti-inflammatory properties. In this review, we provide an overview of the structures and main dietary sources of phenolic acids, encompassing benzoic acid and cinnamic acid. Additionally, we explore the potential of phenolic acids as a nutritional therapy for preventing and treating IBD. In animal and cell experiments, phenolic acids effectively alleviate IBD induced by drug exposure or genetic defects. The mechanisms include improving intestinal mucosal barrier function, reducing oxidative stress, inhibiting excessive activation of the immune response, and regulating the balance of the intestinal microbiota. Our observation points towards the need for additional basic and clinical investigations on phenolic acids and their derivatives as potential novel therapeutic agents for IBD.

## 1. Introduction

Inflammatory bowel disease (IBD) is a chronic intestinal disease with abdominal pain, diarrhea, bloody stool, and other main symptoms, including ulcerative colitis (UC) and Crohn’s disease (CD). CD can occur anywhere in the digestive tract, from the mouth to the anus. It usually involves inflammation of the lining of the digestive tract and can lead to symptoms such as abdominal pain, diarrhea, weight loss, and fatigue. UC mainly affects the colon and rectum. It involves inflammation and ulcers in the lining of the colon, which can cause symptoms such as abdominal pain, diarrhea, and rectal bleeding. IBD is a global condition. Its evolution can be divided into four epidemiological stages: disease emergence, accelerated incidence, disease exacerbation, and disease equilibrium [1]. Western countries are in a worsening phase of the disease, with prevalence rising rapidly and reaching as high as 1% in many areas by 2030. Developing countries are currently in the first stage of disease evolution (the disease emergence stage). The number of IBD patients in China is expected to exceed 1.5 million in 2025 [2,3].

The pathogenesis of IBD is associated with the interactions among genetic, environmental, and immune response factors and the intestinal microbiota, which contributes to the challenge of treating IBD. Currently, the clinical therapeutic repertoire for IBD is predominantly constituted by traditional medications (including aminosalicylic acid preparations, glucocorticoids, and immunosuppressants), biological agents (such as anti-tumor necrosis factor (TNF), interleukin (IL)-12/IL-23), and emerging small-molecule drugs (such as Janus-activated kinase (JAK) inhibitors and sphingosine-1-phosphate (S1P) receptor modulators). However, up to 30% of patients still do not respond to initial treatment, and up to 50% of patients respond over time, and long-term use can lead to many complications [4,5]. These issues prompt us to explore alternative therapies. 

Diet is one of the key factors in the normal intestinal microenvironment, affecting microbial composition, function, the intestinal barrier, and host immunity [6]. In recent years, dietary intervention has emerged as a prominent research focus in both the prevention and treatment of colitis, alongside complementary clinical approaches. Phenolic acid is an important secondary metabolite containing phenolic hydroxyl and carboxylic groups widely distributed in higher plants and possessing various physiological functions such as antibacterial, antioxidant, anti-inflammatory, and anticancer properties. In this review, we summarize the main food sources of phenolic acids, as well as their role and possible mechanisms in the prevention and treatment of colitis. This work aims to delve into understanding how dietary adjustments can regulate the intake of phenolic acids, effectively prevent and treat colitis, and provide valuable information and guidance for the management of related diseases.

## 2. Chemical Structures and Primary Dietary Sources of Phenolic Acids

Phenolic acid refers to organic acids containing phenolic rings, which can be divided into benzoic acids and cinnamic acids according to their structural characteristics (Table 1). 

Phenolic acids are omnipresent in edible vegetables, fruits, and nuts, with an estimated average daily consumption of 1–2 g of these components in a human diet [7]. Salicylic acid, a secondary metabolite associated with plant disease resistance and abiotic stress response, exhibits higher levels in nectarines (3.29 mg/kg), asparagus (1.29 mg/kg), and spices like cumin (29.76 mg/kg), black cumin (25.05 mg/kg), and paprika (28.25 mg/kg) [8]. Protocatechuic acid is more abundant in traditional Chinese medicine and berries such as *Eucommia ulmoides* (1720 mg/100 g), black olives (21 mg/100 g), and black raspberries (8.35 mg/100 g) [9]. Gallic acid is plentiful in plants like rhubarb, eucalyptus, ornus officinalis, and *Chinese pistache* [10]. Vanillic acid is prominent in the roots of Angelica sinensis and is also found in wine, vinegar, and argan oil. Syringic acid, as one of the abundant phenolic compounds, is present in olives, dates, spices, pumpkins, grapes, Brazilian palm trees, honey, red wine, and other plants. Cinnamic acid is found in high concentrations in cinnamon and is also present in citrus fruits, tea, spinach, and celery. p-Coumaric acid is extensively found in fruits (such as apples, pears, grapes, oranges, tomatoes, and berries), vegetables (including legumes, potatoes, and onions), and grains (such as corn, oats, and wheat). It is also present in *Ganoderma lucidum* (1386.4 mg/kg), *Cantharellus cibarius* (2420 mg/kg), lingonberry (85 mg/kg), northern crowberry (122 mg/kg), and sea buckthorn (37 mg/kg) [11]. Sinapic acid is found in fruits, vegetables, grains, oil crops, and certain medicinal plants. In citrus fruits, lemons and Meyer oranges exhibit the highest sinapic acid content, reaching 72.1 μg/g and 50.1 μg/g (dry weight), respectively. Among berries, strawberries and American cranberries demonstrate sinapic acid content reaching 450 μg/g and 210 μg/g, respectively. Caffeic acid is widely present in fruits, vegetables, and beverages, with coffee being a major source. Ferulic acid has a higher content in traditional Chinese medicine, such as Angelic Sinensis Diels (0.05%) and *Cimicifuga foetida* L. (2%), and is also found in daily dietary items, including *Zea mays* (1.65%) and onions (0.32%) [12,13]. Chlorogenic acid, a derivative of hydroxycinnamic acid, is widely found in foods such as coffee, cherries, and apples. It is also a major component in many traditional Chinese herbs, especially honeysuckle and *Eucommia ulmoides* [14]. 

## 3. Effect of Phenolic Acids on Intestinal Mucosal Barrier

The mucosal barrier plays a crucial role in maintaining the stability of the intestinal environment, serving as a protective shield that prevents harmful substances, pathogens, and inflammatory responses from compromising the intestinal tract. In this context, we have summarized the effects of phenolic acid interventions on improving experimental colitis phenotypes, particularly emphasizing improvements in colonic histology and tight junction proteins. The summary of phenolic acids in IBD is shown in Table 2. 

The oral administration of 4-hydroxybenzoic acid (at doses of 10–40 and 100 mg/kg) demonstrated a dose-dependent elevation in tight junction proteins (e-cadherin and occludin) and goblet cell numbers [15,16]. Similarly, treatment with 4-hydroxybenzoic acid (at concentrations of 3, 10, and 30 μM) resulted in an increased expression of tight junction proteins in mice and Caco-2 cells exposed to TNF-α (10 ng/mL) [15]. 

Protocatechuic acid, administered at doses of 5, 10, and 20 mg/kg, effectively alleviated the dextran sulfate sodium salt (DSS)-induced reduction in occludin protein expression in mice treated with 3.5% DSS [17]. Furthermore, intraperitoneal administration of protocatechuic acid at doses of 10, 30, or 60 mg/kg per day prevented both the macroscopic and microscopic damage to the colonic mucosa, as well as the decline in body weight gain in mice treated with 2,4,6-trinitrobenzene sulfonic acid (TNBS) and rats treated with DSS [18,19]. 

Gallic acid, administered at doses ranging from 10 to 200 mg/kg, demonstrated a significant attenuation of the disease activity index, colon shortening, and reduction in histopathological evidence of injury [20,21,22,23,24]. Additionally, gallic acid upregulated the expression of nuclear factor erythroid 2-related factor 2 (NRF2) and mitigated the activation and nuclear accumulation of p-STAT3. As a result, this prevented the degradation of the inhibitory protein IκB and hindered the nuclear translocation of p65-nuclear factor kappa-B (NF-κB) in the colonic mucosa [22,23].

Vanillic acid, provided at a dose of 4000 mg/kg in the diet, elevated the expression of the tight junction protein occludin in a weaned piglet model challenged with 10 mg/kg lipopolysaccharides (LPS) [25]. Additionally, at a dose of 200 mg/kg, vanillic acid mitigated DSS-induced body weight loss and colon shortening [26]. 

Syringic acid, administered at concentrations of 0.1, 1.0, and 10.0 μM, effectively mitigated the disruption of the intestinal barrier in Caco-2 cells in response to oxygen–glucose deprivation/reoxygenation (OGD/R). This protective effect was assessed through the preservation of intestinal epithelial integrity and the modulation of protein expression levels, including claudin-3, claudin-2, and ZO-1 [27]. Additionally, in mice with DSS-induced colitis, syringic acid at doses of 25 and 50 mg/kg prevented colon damage, alleviated proptosis, and increased the mRNA expression of intestinal barrier proteins (claudin-1, ZO-1, and occludin) [28,29]. Furthermore, treatment with 25 and 50 mg/kg of syringic acid decreased the mean macroscopic ulcer score in rats with acetic acid-induced colitis [30]. 

Cinnamic acid, administered at doses of 20, 30, and 50 mg/kg, ameliorated the histological assessment of colon tissue in mice with DSS-induced colitis and rats with dinitrobenzene sulfonic acid (DNBS)-induced colitis [31,32]. 

Coumaric acid, at doses of 100 and 150 mg/kg, exhibited a positive impact on the macroscopic changes in the colons of rats with acetic acid-induced colitis [30]. 

Caffeic acid, administered at doses of 50 and 250 mg/kg, mitigated the disease severity in mice with DSS-induced colitis [33,34]. 

Ferulic acid, provided at doses of 20 and 40 mg/kg, not only alleviated TNBS-induced ulcerative colitis but also inhibited cell apoptosis, as evidenced by the modulation of caspase-1 and caspase-3 expression in rats [35,36]. 

Sinapic acid, administered at doses of 10, 30, and 100 mg/kg, demonstrated improvement in the macroscopic changes associated with TNBS-induced colitis in mice. This improvement was evidenced by morphological observations of the mouse colon, measurements of colon length, weight, and the colon weight/length ratio, as well as macroscopic scoring [37]. Additionally, sinapic acid exhibited a positive effect on colonic claudin-1, occludin, and ZO-1 in Kunming mice with DSS-induced colitis [38]. Furthermore, sinapic acid impeded the impairment of intestinal permeability and the redistribution of tight junction proteins in differentiated Caco-2 cells provoked by 20 μg/mL LPS and 20 ng/mL TNF-α. Moreover, it suppressed the myosin light chain kinase (MLCK)/myosin light chain (MLC)/NF-κB signaling pathways and the phosphorylation of activating transcription factor 2 (ATF-2) in response to stimulus-induced conditions in differentiated Caco-2 cells [39,40]. 

Chlorogenic acid, at a concentration of 1 mM, mitigated various effects of DSS-induced colitis, including weight loss and increased disease activity [41]. Additionally, treatment with chlorogenic acid at a dose of 20 mg/kg alleviated mucosal damage induced by both DSS and TNBS, operating through the MAPK/ERK/JNK signaling pathway [42,43]. Furthermore, the administration of chlorogenic acid at a dose of 50 mg/kg protected against body weight loss, preserved intestinal morphology, and maintained integrity in mice treated with indomethacin [44].

## 4. Effect of Phenolic Acids on Oxidative Stress

Oxidative stress is characterized by an imbalance in cellular oxidative status due to an excess of free radicals and oxidizing agents, potentially leading to cellular damage and inflammation. Phenolic acids are considered to play a positive role in modulating oxidative stress in the intestines due to their antioxidant properties.

When administered intraperitoneally at doses of 10, 30, or 60 mg/kg/day, protocatechuic acid successfully averted both visible and microscopic harm to the colonic mucosa. Furthermore, it alleviated the decline in body weight gain and the rise in myeloperoxidase activity triggered by TNBS-treated mice and DSS-treated rats [18,19]. Moreover, mice subjected to protocatechuic acid treatment exhibited a decreased ratio of oxidized to reduced glutathione, coupled with elevated levels of antioxidant enzymes and Nrf2 expression amidst TNBS-induced colitis [18]. 

Syringic acid, at concentrations of 0.1, 1.0, and 10.0 μM, effectively mitigated oxidant stress in oxygen–glucose deprivation/reoxygenation-induced Caco-2 cells, including the levels of reactive oxygen species (ROS), malondialdehyde (MDA), and superoxide dismutase (SOD), and attenuated apoptosis [27]. In the context of DSS-induced colitis, syringic acid at a dose of 25 mg/kg reduced the activity of MDA, glutathione (GSH), and SOD in the colon [28,29]. Additionally, treatment with 25 and 50 mg/kg of syringic acid increased the mRNA expression of heme oxygenase-1 (Ho-1), NAD(P)H: quinone acceptor oxidoreductase 1 (NQO1), and NRF2 in colon tissue in acetic acid-induced colitis rats [30].

Coumaric acid demonstrated an improvement in oxidative stress, including SOD and total antioxidant capacity (TAC), induced by acetic acid-induced colitis in the colon [45]. Furthermore, at doses of 100 and 150 mg/kg, coumaric acid increased the mRNA expression of HO-1, NRF2, and NQO1 in acetic acid-induced rats [30]. 

Caffeic acid treatment resulted in a significant decrease in MDA levels and an increase in TAC, SOD, glutathione peroxidase (GSH-PX), and catalase (CAT) in serum [34]. 

Ferulic acid, administered at doses of 20, 40, and 60 mg/kg, significantly increased the activity of antioxidant factors, including TAC content and SOD and CAT activity, in the colon tissue of rats with acetic acid-induced colitis [46]. 

Animals treated with sinapic acid at a dose of 40 mg/kg exhibited a noteworthy replenishment in mean CAT and GSH levels compared to those with acetic acid-induced colitis, indicating a restoration of free radical scavenging activity [47]. 

Chlorogenic acid, at a dose of 20 mg/kg, ameliorated DSS-induced oxidative stress in the colon [43].

## 5. Effect of Phenolic Acids on Immune System

The immune system plays a pivotal role in the intestines by safeguarding the body against external pathogens and harmful substances. Phenolic acids are recognized for their anti-inflammatory properties, effectively mitigating inflammatory responses and thereby preserving the equilibrium of the intestinal immune system [48]. Furthermore, phenolic acids have the potential to modulate the activity and differentiation of immune cells, thereby influencing the regulation of immune responses. This regulatory impact serves to prevent undue immune activation and uphold the normal functionality of the immune system.

Oral administration of 4-hydroxybenzoic acid, at doses ranging from 10 to 100 mg/kg, exhibited a dose-dependent attenuation of inflammatory cytokine levels, including IL-6, TNF-α, and IL-1β [15,16]. Additionally, treatment with 4-hydroxybenzoic acid at concentrations of 3, 10, and 30 μM decreased the expression of proinflammatory cytokines in both mice and Caco-2 cells treated with TNF-α (10 ng/mL) [15]. 

Oral administration of protocatechuic acid at doses of 5, 10, and 20 mg/kg suppressed the DSS-induced increase in inflammatory factors, including IL-6, IL-12, and TNF-α, in mice treated with 3.5% DSS [17]. Furthermore, protocatechuic acid-treated mice exhibited a reduction in the expression of proinflammatory cytokines (IL-6, IL-1β, TNF-α, and cyclooxygenase 2 (COX-2)) in TNBS-induced colitis, achieved by modulating the sphingosine kinase (SphK)/S1P and related signaling pathways [18].

Gallic acid, administered at doses of 10 mg/kg or 40, 80, and 120 mg/kg, significantly reduced the mRNA expressions of IL-21, IL-23, TNF-α, IL-1β, IL-6, and IL-17 in the serum and colon tissue of DSS-induced mice [20,21,22]. Moreover, the administration of gallic acid at doses of 20, 40, and 60 mg/kg significantly increased the expressions of IL-4 and IL-10 while downregulating IL-1, IL-6, IL-12, IL-17, IL-23, transforming growth factor-β (TGF-β), and TNF-α in the colon tissues of TNBS-induced colitis mice and IL-1β-induced HIEC-6 cells [23]. Gallic acid significantly reduced myeloperoxidase (MPO) activity, inducible nitric oxide synthase (iNOS), and cyclooxygenase (COX)-2 [22,23]. 

Vanillic acid, provided at a dose of 4000 mg/kg in the diet, decreased serum levels of IL-1β, IL-2, IL-6, and TNF-α in a weaned piglet model challenged with 10 mg/kg LPS [25]. Additionally, vanillic acid at a dose of 200 mg/kg reduced the level of IL-6 in the plasma of DSS-treated mice [26]. 

Syringic acid, at concentrations of 0.1, 1.0, and 10.0 μM, effectively suppressed the release of inflammatory cytokines in Caco-2 cells subjected to oxygen–glucose deprivation/reoxygenation, which includes IL-6, IL-1β, and monocyte chemoattractant protein-1 (MCP-1) [27]. Additionally, syringic acid at doses of 25 and 50 mg/kg alleviated the inflammatory response, including TNF-α, IL-1β, and IL-6, in both the colon and serum. It also reduced the expression of p65-NFκB, iNOS, and COX-2 in mice with DSS-induced colitis [28,29]. Furthermore, treatment with 10, 25, and 50 mg/kg of syringic acid significantly decreased the tissue levels of TNF-α and IL-1β proteins compared to the UC group in rats with acetic acid-induced colitis [30].

Cinnamic acid administration at doses of 25 and 50 mg/kg resulted in reduced levels of TNF-α and IL-6 in mice with DSS-induced colitis [31].

Coumaric acid modulated the expression of inflammatory markers, including NF-κB, TNF-α, iNOS, IL-1β, and IL-6, in the colon during acetic acid-induced colitis [30,45]. 

Caffeic acid suppressed the production of inflammatory cytokines (TNF-α, IL-6, IL-12, and IL-1β) in the colon and interfered with the infiltration and function of mononuclear macrophages in the mucosa, mediastinal lymph nodal, and spleens of DSS-induced mice [33,34,49]. 

Ferulic acid, administered at doses of 20, 40, and 60 mg/kg, significantly inhibited the mRNA expression of inflammatory and apoptotic genes in the colon tissue of rats with acetic acid-induced colitis [46]. Additionally, at doses of 20 and 40 mg/kg, ferulic acid ameliorated TNBS-induced colitis by inhibiting the production of proinflammatory cytokines (TNF-α, IL-1β, IL-6, AND IL-10) and downregulating COX-2 synthesis [35,36]. Moreover, ferulic acid treatment restored the viability of human intestinal microvascular endothelial cells (HIMEC) and inhibited TNF-α-induced cell inflammation [35]. 

Sinapic acid, administered at doses of 10, 30, and 100 mg/kg, improved the changes in the expression of inflammation mediators in mice with TNBS-induced colitis. This enhancement was evidenced by decreased MPO activity, MDA levels, and TNF-α production in colonic tissues [37]. Additionally, at a dose of 40 mg/kg, sinapic acid demonstrated a significant decline in mean TNF-α and IL-6 levels in mice with acetic acid-induced colitis [47]. Also, sinapic acid reduces serum levels of proinflammatory cytokines (TNF-α, IL-1β, IL-6, IL-17α, IL-18, and interferon (IFN)-γ) and increases anti-inflammatory cytokines (IL-4 and IL-10) in Kunming mice with DSS-induced colitis [38]. Moreover, sinapic acid, administered at doses of 10 and 50 mg/kg, notably inhibited the mRNA expression of proinflammatory cytokines IL-1β, IL-6, and TNF-α in differentiated Caco-2 cells stimulated with 20 μg/mL LPS and 20 ng/mL TNF-α. This inhibition was accomplished by attenuating the activation of the Toll-like receptor 4 (TLR4)/NF-κB pathway [39,40]. 

Chlorogenic acid, at concentrations of 1 mM or 20 mg/kg, markedly reduced the release of IFN-γ, TNF-α, and IL-6 and the infiltration of F4/80+ macrophages, CD3+ T cells, and CD177+ neutrophils into the colon by blocking the activated NF-κB signaling pathway in DSS-induced colitis [41,42,43]. Moreover, at a dose of 50 mg/kg, chlorogenic acid prevented inflammation (TNF-α, IL-1β, and IL-6) in the colons of indomethacin-treated mice [44]. Additionally, at a dose of 1 mg/kg, chlorogenic acid significantly increased the ratio of CD4+/CD8+ T cell subsets in Peyer’s patches and mesenteric lymph nodes while reducing the expression levels of iNOS, TNF-α, and IL-1β [50]. Furthermore, chlorogenic acid, at concentrations of 0.5, 1, and 2 mM, reduced TNF-α and H_2_O_2_-induced IL-8 production in Caco-2 cells [51]. Chlorogenic acid, ranging from 15.63 to 250 μM, markedly improved cellular vitality based on LPS/ATP-treated RAW264.7 cells and notably restrained the production of IL-1β through the NF-κB/NLR family pyrin domain containing 3 (NLRP3) pathway [52]. Finally, chlorogenic acid alleviated colitis by decreasing M1 macrophage polarization via the inhibition of pyruvate kinase M2-dependent glycolysis and the suppression of NLRP3 activation [53].

## 6. Effect of Phenolic Acids on Gut Microbes

The gut microbiota plays a crucial role in maintaining intestinal health and immune balance [54]. Phenolic acids may impact the composition of the gut microbiota through their antioxidant and anti-inflammatory properties. This influence may result in an increase in beneficial bacteria and a decrease in harmful bacteria, thereby promoting a balance in the gut microbial ecosystem. Phenolic acids may create a favorable environment for probiotics, enhancing their survival and functionality in the gut. This promoting effect contributes to maintaining the diversity and stability of the gut microbiota, exerting a positive impact on overall intestinal health.

Oral administration of 4-hydroxybenzoic acid (100 mg/kg) effectively relieved the DSS-induced colitis in mice, which were largely dependent on the gut microbiota, as antibiotic treatment substantially attenuated the improvement of colitis by 4-hydroxybenzoic acid, and transplantation of gut microbiota from colitis mice treated with 4-hydroxybenzoic acid significantly reduced the colitis. The transplantation of colitis mice treated with HA increased the abundance of *Akkermansia muciniphila* [16]. 

Protocatechuic acid, at a dose of 20 mg/kg, regulated the composition of the gut microbiota. In the DSS group, the relative abundances of *Bacteroidetes* and *Verrucomicrobiota* decreased compared to the control group. The treatment with protocatechuic acid partially restored their relative abundances [17].

Gallic acid, administered at a dose of 200 mg/kg, improved gut microbiota dysbiosis induced by DSS. Further fecal microbiota transplantation demonstrated that gallic acid’s anti-colitis effects were mediated by the gut microbiota. At the taxonomic level, gallic acid replenished the presence of all primary phyla except Actinobacteria. Regarding order-level taxonomy, treatment with gallic acid replenished the presence of *Clostridiales*, *Enterobacterales,* and *Bacteroidales*. Moreover, among the genera identified as differentially abundant by the linear discriminant analysis effect size algorithm, the gallic acid group exhibited enrichment in *Erysipelatoclostridium* and *Eubacterium* [24].

Vanillic acid elevated the Firmicutes/Bacteroidetes ratio while diminishing the proportional presence of *Prevotellaceae*. It additionally bolstered the *Lachnospiraceae* family, particularly the *Lachnospiraceae* FCS020 group. Furthermore, vanillic acid decreased the relative occurrence of *Prevotella 7* and *Prevotella 1* but enhanced *Lachnospira*, the *Eubacterium eligens* group, and the *Eubacterium xylanophilum* group in a weaned piglet model exposed to a 10 mg/kg LPS challenge [25]. 

Research on syringic acid has indicated its potential similarity to the impact of transplanted healthy mouse feces in addressing DSS-induced colitis [29]. 

Supplementation with caffeic acid (250 mg/kg) altered the composition of the gut microbiome by decreasing the relative abundance of *Bacteroides* and *Turicibacter* while enhancing the relative abundance of *Alistipes* and *Dubosiella* in DSS-induced colitis [34]. 

Oral administration of sinapic acid (2 and 10 mg/kg) alleviated DSS-induced IBD and modified the gut microbiota. At the genus level, sinapic acid was found to inhibit DSS-induced declines in the relative abundance of *Ligilactobacillus* and *Limosilactobacillus* in the feces of C57BL/6J mice, and it suppressed the DSS-induced decrease in the Firmicutes/Bacteroidetes (F/B) ratio [39]. 

Chlorogenic acid (1 mM) decreased the proportion of Firmicutes and Bacteroidetes and increased the proportion of the mucin-degrading bacterium *Akkermansia* in DSS-induced colitis mice [41]. Moreover, chlorogenic acid (2% in the diet) reversed the decrease in diversity caused by DSS and improved the relative abundance of organisms in the genus Lactobacillus [55]. Additionally, at a dose of 50 mg/kg, chlorogenic acid inhibited the growth of *Bacteroides* and the accumulation of *Bacteroides*-derived LPS in indomethacin-induced colitis [44].

## 7. Conclusions and Future Perspectives 

Numerous studies have focused on the potential therapeutic effects of phenolic acid compounds on colitis and their potential roles in the prevention and treatment of this inflammatory gastrointestinal disease. Some studies suggest that phenolic acids possess anti-inflammatory and antioxidant properties, which may play a beneficial role in alleviating inflammation and oxidative stress associated with colitis. These compounds are believed to influence the development and progression of colitis by mitigating inflammatory responses, regulating the immune system, and protecting the mucosal barrier.

However, despite some promising research findings, there is currently a lack of comprehensive studies and a thorough understanding of the mechanisms of action regarding the role of phenolic acids in colitis treatment. Researchers are actively working to delve deeper into questions related to the molecular mechanisms of phenolic acids, their applicability across different disease stages, and the optimal intake levels. These efforts aim to provide a more comprehensive assessment of the potential role of phenolic acids in the management of colitis.

## Figures and Tables

**Table 1:** Chemical structure of phenolic acids.

	Phenolic Compounds	Substituent Group
Benzoic acid 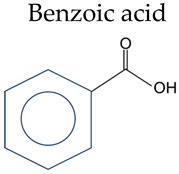	Salicylic acid	2-OH
	3-Hydroxybenzoic acid	3-OH
	4-Hydroxybenzoic acid	4-OH
	2,3-Dihydroxybenzoic	2,3-OH
	Gentisic acid	2,5-OH
	Protocatechuic acid	3,4-OH
	Gallic acid	3,4,5-OH
	Vanillic acid	4-OH, 3-OCH_3_
	Isovanillic Acid	3-OH, 4-OCH_3_
	Syringic	4-OH, 3,5-OCH_3_
Cinnamic acid 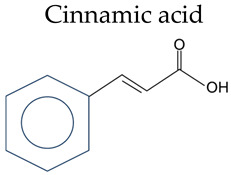	Cinnamic acid	/
	o-Coumaric acid	2-OH
	p-Coumaric acid	4-OH
	Caffeic acid	3,4-OH
	Ferulic Acid	4-OH, 3-OCH_3_
	Sinapic acid	4-OH, 3,5-OCH_3_
	Chlorogenic acid	3,4-OH, 1-Quinic

**Table 2:** Summary of phenolic acids on IBD.

Phenolic Acid Compounds	Model	Dose	Effect	Reference
4-Hydroxybenzoic acid	2.5% DSS treated mice for 7 days	10–40 mg/kg and 100 mg/kg, orally	reduced inflammatory cytokines; improved mucosal barrier	Xu X [15], Han X [16]
	10 ng/mL TNF-α treated Caco-2 cells	3, 10 and 30 μM	decreased the expression of proinflammatory cytokines; increased the expression of tight junction proteins	Xu X [15]
Protocatechuic Acid	3.5% DSS treated C57BL/6 mice for 7 days	5, 10, and 20 mg/kg, orally	reduced inflammatory factors; increased occluding protein expression	Yang X [17]
	20 mg/mL of TNBS-treated BALB/c mice through the catheter	30 and 60 mg/kg, intraperitoneally	prevented the macroscopic damage and the increase in myeloperoxidase activity; increased expression of antioxidant enzymes; reduced expression of proinflammatory cytokines	Crespo I [18]
	5% DSS treated rat for 5 days	10 mg/kg, orally	prevented the increase in proinflammatory cytokines in the plasma; suppressed the DSS-mediated elevation in colonic myeloperoxidase activity	Farombi EO [19]
Gallic acid	2.5% DSS treated BALB/c mice for 7 days	10 mg/kg, orally	reduced inflammation; improved oxidative stress; upregulated the expression of Nrf2 and its downstream targets	Pandurangan AK [20]
	2.5% DSS treated BALB/c mice for 7 days	10 mg/kg, orally	reduced the expression of inflammatory mediators; suppressed p65-NF-κB and IL-6/p-STAT3 activation	Pandurangan AK [21]
	TNBS treated BALB/c mice through the catheter.	20, 40, 60 mg/kg, orally	reduced inflammation; suppressed NF-κB	Zhu L [22]
	10 ng/mL IL-1β treated HIEC-6 cells	20, 40, 60 mg/kg	inhibited apoptosis	Zhu L [22]
	3.5% DSS treated BALB/c mice for 7 days	40, 80, 120 mg/kg, orally	reduced inflammation; downregulated the expressions of NLRP3	Yu T-Y [23]
	2.5% DSS treated C56B/6L mice for 10 days	200 mg/kg, orally	trapped deleterious metabolite ammonia; improved gut microbiota dysbiosis	Peng J [24]
Vanillic acid	10 mg/kg LPS-treated weaned piglet	4000 mg/kg in diet	decreased serum levels of proinflammatory factor; enhanced the expression of tight junction protein; modulated gut microbiota	Hu R [25]
	5% DSS treated BALB/c mice for 7 days	200 mg/kg, orally	relieved colitis; reduced IL-6	Kim S-J [26]
Syringic Acid	OGD/R-stimulated cell injury in Caco-2 cell	0.1, 1.0 and 10.0 μM	inhibited intestinal barrier disruption; ameliorated apoptosis; attenuated oxidant stress; suppressed the release of inflammatory cytokines	Xiang S [27]
	3.5% DSS treated BALB/c mice for 7 days	25 mg/kg, orally	suppressed proinflammatory cytokine; prevented DSS-induced colon damage; reduced the activity of MPO	Fang W [28]
	2.5% DSS treated C56BL/6 mice for 7 days	50 mg/kg, orally	regulated oxidative stress; alleviated inflammatory response; relieved proptosis	Luo Q [29]
	0.8 mL of 7% acetic acid was instilled into the rat colon through the cannula	10, 25, and 50 mg/kg	decreased the mean macroscopic ulcer score; increased HO-1, Nrf2, and NQO1 mRNA expression; decreased the tissue levels of TNF-α and IL-1β	Ekhtiar M [30]
Cinnamic acid	2.5% DSS treated albino mice for 7 days	25 and 50 mg/kg, orally	reduced the levels of TNF-α and IL-6	Habboby M [31]
	0.4 mL 120 mg/mL DNBS was instilled into the rat colon through the cannula	30 mg/kg, orally	activated GPR109A in the inflamed colon	Kang C [32]
Coumaric acid	0.8 mL 7% acetic acid was rectally injected into rats	50, 100, and 150 mg/kg, orally	improved oxidative stress; improved the inflammation	Ghasemi-Dehnoo M [33]
	0.8 mL 7% acetic acid was rectally injected into rats	100 and 150 mg/kg	decreased the mean macroscopic ulcer score; increased HO-1, Nrf2, and NQO1 mRNA expression; decreased the tissue levels of TNF-α and IL-1β	Ekhtiar M [30]
Caffeic acid	3.5% DSS treated C57BL mice t for 7 days	50 mg/kg, orally	suppressed the production of inflammatory cytokines; interfered with the infiltration and function of mononuclear macrophages	Xiang C [34]
	3.5% DSS treated ICR mice for 7 days	250 mg/kg, orally	decreased proinflammatory cytokines; increased the level of IL-10; altered the gut microbiome composition	Wan F [35]
	1 ng/mL IL-1β treated CCD-18Co cells	10 and 50 μM	reduced the biosynthesis of IL-8 and MCP1,	Zielinska D [36]
Ferulic acid	0.8 mL 7% acetic acid was rectally injected into rats	20, 40, and 60 mg/kg, orally	inhibited inflammatory, apoptotic, and production of MDA and NO; increased the activity of antioxidant factors	Ghasemi-Dehnoo M [37]
	1% DSS treated C57BL mice t for 16 days	50 mg/kg, orally	improved histopathologic score and MPO activity	Islam MS [38]
	100 mg/kg TNBS was rectally injected into rats	20 and 40 mg/kg, orally	suppression of oxidative stress, apoptosis, production of proinflammatory cytokines, and inhibition of COX-2 synthesis	Sadar SS [39]
	100 mg/kg TNBS was rectally injected into rats	10, 20 and 250 mg/kg, orally	inhibited the inflammatory injury of endothelial cells;	Yu S [40]
	10 ng/mL TNF-α treated HIMECs	125, 250, 500 μM	reduced the expression of inflammatory factors; improved cell viability	Yu S [40]
Sinapic acid	30 mg/kg TNBS was rectally injected into BABL/c mice	10, 30, and 100 mg/kg, orally	improved the macroscopic changes of colonic damage; improved the changes in expression of biochemical mediators of inflammation	Lee JY [41]
	20 μg/mL LPS and 20 ng/mL TNF-α treated Caco-2 cells	12.5, 25 and 50 μM	suppressed impairment of intestinal permeability and cellular reorganization of tight junction proteins	Jang S [42]
	2% DSS treated C57BL mice for 7 days	2 and 10 mg/kg, orally	alleviated DSS-induced IBD; modified gut microbiota	Jang S [42]
	20 μg/mL LPS-treated Caco-2 cells	5, 10 and 15 μM	reduced the expression of proinflammatory cytokines; improved tight junction mRNA levels	Lan H [43]
	2 mL 4% acetic acid was rectally injected into rats	40 mg/kg, orally	suppressed inflammation, oxidative stress, and apoptosis	Shahid M [44]
	2% DSS treated Kunming mice for 7 days	10 and 50 mg/kg, orally	attenuated intestinal permeability; reduced inflammatory; attenuated oxidative damage; reduced the activation of the NLRP3 inflammasome	Qian B [45]
Chlorogenic acid	2.5% DSS treated C57BL/6 mice for 8 days	1 mM, orally	suppressed inflammation; modified gut microbiota; promoted the growth of *Akkermansia*	Zhang Z [46]
	BALB/c mice intracolonic administration of 4 mg in 0.1 mL of 30% ethanol TNBS	20 mg/kg, orally	anti-inflammatory; decreased neutrophil infiltration and suppression of NF-κB-dependent pathways.	Zatorski H [47]
	5% DSS treated C57BL/6 mice for 7 days	30, 60, and 120 mg/kg, orally	reduced mucosal damage; inhibited colonic mucosal inflammation; improved colitis through MAPK/ERK/JNK signaling pathway	Gao W [48]
	2 mM H_2_O_2_ and 10 ng/mL of TNF-α treated Caco-2 cells	0.5, 1 and 2 mM	reduced IL-8 secretion	Shin HS [49]
	3% DSS treated C57BL/6 mice for 8 days	1 mM, orally	reduced proinflammatory cytokines	Shin HS [49]
	2.5% DSS treated C57BL/6 mice for 5 days	2% in diet	decreased the production of proinflammatory cytokines; and restored intestinal microbial diversity.	Zhang P [50]
	5 mg/kg indomethacin treated C57BL/6 mice for 5 days	50 mg/kg, orally	prevented inflammation; prevented intestinal barrier dysfunction; decreased *Bacteroides*-derived LPS	Yan Y [51]
	IL-10 KO mice	1 mg/kg, orally	increased the ratio of CD4+/CD8+ T cell subsets; prevented inflammation	Lee YM [52]
	3% DSS treated BALB/c mice for 7 days	20 and 40 mg/kg, orally	prevented inflammation	Zeng J [53]
	0.5 ug/mL LPS and 2 nM ATP induced Raw264.7 cells	15.63, 31.25, 62.5, 125 and 250 μM	improved the cellular vitality	Zeng J [53]

DSS, dextran sulfate sodium salt; TNF-α, tumor necrosis factor alpha; TNBS, 2,4,6-trinitrobenzene sulfonic acid; Nrf2, nuclear factor erythroid 2-related factor 2; NF-κB, nuclear factor kappa-B; IL, interleukin; NLRP3, NLR family pyrin domain containing 3; LPS, lipopolysaccharides; OGD/R, oxygen–glucose deprivation/reoxygenation; MPO, myeloperoxidase; HO-1, heme oxygenase-1; DNBS, dinitrobenzene sulfonic acid; MCP1, monocyte chemoattractant protein-1; MDA, malondialdehyde; COX-2, cyclooxygenase 2.
